# Can Dynamic Imaging, Using ^18^F-FDG PET/CT and CT Perfusion Differentiate Between Benign and Malignant Pulmonary Nodules?

**DOI:** 10.2478/raon-2021-0024

**Published:** 2021-05-31

**Authors:** Aleksander Marin, John T. Murchison, Kristopher M. Skwarski, Adriana A.S. Tavares, Alison Fletcher, William A. Wallace, Vladka Salapura, Edwin J.R. van Beek, Saeed Mirsadraee

**Affiliations:** 1 Edinburgh Imaging facility Queens Medical Research Institute, University of Edinburgh, Edinburgh, United Kingdom; 2 Faculty of Medicine, University of Ljubljana, Ljubljana, Slovenia; 3 Department of Radiology, Royal Infirmary of Edinburgh, Edinburgh, United Kingdom; 4 Department of Respiratory Medicine, Royal Infirmary of Edinburgh, Edinburgh, United Kingdom; 5 Department of Pathology, Royal Infirmary of Edinburgh, Edinburgh, United Kingdom; 6 National Heart and Lung Institute, Imperial College London, London, United Kingdom

**Keywords:** pulmonary nodule, perfusion, CT, dynamic, PET/CT

## Abstract

**Background:**

The aim of the study was to derive and compare metabolic parameters relating to benign and malignant pulmonary nodules using dynamic 2-deoxy-2-[fluorine-18]fluoro-D-glucose (^18^F-FDG) PET/CT, and nodule perfusion parameters derived through perfusion computed tomography (CT).

**Patients and methods:**

Twenty patients with 21 pulmonary nodules incidentally detected on CT underwent a dynamic ^18^F-FDG PET/CT and a perfusion CT. The maximum standardized uptake value (SUV_max_) was measured on conventional ^18^F-FDG PET/CT images. The influx constant (*K_i_*) was calculated from the dynamic ^18^F-FDG PET/CT data using Patlak model. Arterial flow (AF) using the maximum slope model and blood volume (BV) using the Patlak plot method for each nodule were calculated from the perfusion CT data. All nodules were characterized as malignant or benign based on histopathology or 2 year follow up CT. All parameters were statistically compared between the two groups using the nonparametric Mann-Whitney test.

**Results:**

Twelve malignant and 9 benign lung nodules were analysed (median size 20.1 mm, 9–29 mm) in 21 patients (male/female = 11/9; mean age ± SD: 65.3 ± 7.4; age range: 50–76 years). The average SUV_max_ values ± SD of the benign and malignant nodules were 2.2 ± 1.7 *vs*. 7.0 ± 4.5, respectively (p = 0.0148). Average *K_i_* values in benign and malignant nodules were 0.0057 ± 0.0071 and 0.0230 ± 0.0155 min^-1^, respectively (p = 0.0311). Average BV for the benign and malignant nodules were 11.6857 ± 6.7347 and 28.3400 ± 15.9672 ml/100 ml, respectively (p = 0.0250). Average AF for the benign and malignant nodules were 74.4571 ± 89.0321 and 89.200 ± 49.8883 ml/100g/min, respectively (p = 0.1613).

**Conclusions:**

Dynamic ^18^F-FDG PET/CT and perfusion CT derived blood volume had similar capability to differentiate benign from malignant lung nodules.

## Introduction

Pulmonary nodules are detected with increasing frequency due to widespread use of computed tomography (CT).^1^,^2^ The prevalence of incidental pulmonary nodules on standard CT studies is around 13%, while lung cancer screening will detect lung nodules in up to 53% of subjects, leading to a lung cancer prevalence of around 1.4% (0.5–2.7%).^3^ The optimal diagnostic approach for the management of indeterminate pulmonary nodules has been the subject of much discussion.^4^

The widely accepted guidelines published by the British Thoracic Society (BTS) and the Fleischner Society recommend the minimum nodule diameter thresholds and CT follow-up time intervals for surveillance of solitary nodules smaller than 8 mm.^3^,^5^ For nodules of ≥ 8 mm (300 mm^3^), the BTS guidelines recommend risk assessment using the Brock model. The above guidelines recommend either 3-month CT follow-up, work-up with positron emission tomography (PET) with 2-deoxy-2-[fluorine-18]fluoro-D-glucose (18F-FDG), tissue sampling, or resection for nodules of ≥ 8mm. CT characterisation using only morphological features is imprecise^6^,^7^, leading to an increased interest in computer-based radiomics assessment.^8^, ^9^, ^10^, ^11^, ^12^, ^13^, ^14^, ^15^ Serial CT imaging to monitor nodule size can be problematic as nodule growth varies with different cancers and causes patient anxiety.^16^, ^17^, ^18^ ^18^F-FDG PET has high sensitivity but lower specificity of 82% for detecting malignant pulmonary nodules, particularly in those smaller than 10 mm.^19^ Imaging guided sampling of small nodules is also difficult, is associated with complications, and its diagnostic yield decreases further as nodule size decreases.^3^,^20^,^21^

Neovascularisation is a complex process known to be central to carcinogenesis.^22^ Advances in the imaging technology in the last two decades have enabled the study of perfusion characteristics within pulmonary nodules.^23^, ^24^, ^25^, ^26^, ^27^ As benign and malignant lesions have different vascularity, different perfusion parameters and dynamic ^18^F-FDG uptake properties can be expected.^27^, ^28^, ^29^, ^30^, ^31^, ^32^

The purpose of this pilot study was to evaluate the feasibility and accuracy of CT perfusion and dynamic ^18^F-FDG PET imaging in differentiating proven benign and malignant pulmonary nodules.

## Patients and methods

This single-centre prospective study was approved by the local Research Ethics Committee (13/SS/0153) and written informed consent was obtained from all participants.

Between December 2014 and December 2015, 20 consecutive patients who were referred to our respiratory outpatient clinic for an indeterminate incidental pulmonary nodule were recruited. The inclusion criteria were: a) incidentally detected soft tissue (solid) pulmonary nodules measuring ≥ 8 mm and < 30 mm on CT, b) either surgical excision, imaging guided biopsy or imaging follow up of the nodule planned. The exclusion criteria were: a) abnormal renal function, b) previous adverse reaction to iodinated contrast agent, c) known history of malignancy, d) pregnancy or breast feeding, e) patients who refused or were unable to provide informed consent.

The patients underwent a dynamic ^18^F-FDG PET/ CT and dynamic perfusion CT imaging within a 3 week time frame (mean, 6.4 days: range 1–18 days). Due to technical reasons, the dynamic PET data could not be used in 4 patients for the analysis, one of these patients had two synchronous nodules. CT perfusion analysis was performed in 17 of the nodules. One patient declined the CT perfusion scan and 3 patients had significant breathing artefact on the scans, rendering analysis non-feasible. All nodules were classified into either benign or malignant on the basis of a histopathological diagnosis (n = 16), or stability during 2 years follow up CT imaging (n = 5).

### Dynamic PET/CT image acquisition

All patients were fasted for at least six hours before the imaging. Following a low dose CT scan for attenuation correction and localisation (120 kV, 50 mAs, 5/3 mm), patients were administered 400 MBq of ^18^F-FDG intravenously, and a dynamic 60 minute image acquisition was performed using a Siemens Biograph PET/CT scanner (Siemens Healthcare, Erlangen, Germany). Respiratory-gated PET data were reconstructed using a 15-frame protocol (7 frames×180 s, 7×300 s, 1×240 s), a matrix size of 256×256×53 with a voxel size of 2.65×2.65×3.00 mm^3^, and subsets expectation maximization (OSEM) method. A conventional PET/CT scan was performed on completion of the dynamic phase of the scan at 1 hour after injection of the tracer.

### Perfusion volume CT acquisition

Dynamic perfusion CT scans were performed as previously described 25,28,33,34 on a 320-detector row CT scanner (Aquilion ONE; Toshiba Medical Systems, Tokyo, Japan) with 16 cm field of view coverage. Imaging was performed at 0, 2, 4, 6, 8, 10, 12, 14, 16, 18, 20, 24, 30, 40, 50, 60, 90, 120 seconds, 3 minutes, 4 minutes, and 10 minutes following the intravenous injection of 70 ml of iodinated contrast (Iomeron 400 mg/ml, Bracco, Milan, Italy) followed by a 30 ml bolus of saline both at 9 ml/s through a 16 G cannula sited in the ante cubital fossa. Acquisition parameters were 100 kV, 100 mA, 0.5 seconds rotation time, 320 x 0.5 mm collimation, 512 x 512 matrix.

### Image analysis

#### Dynamic PET/CT

**Figure 1:**
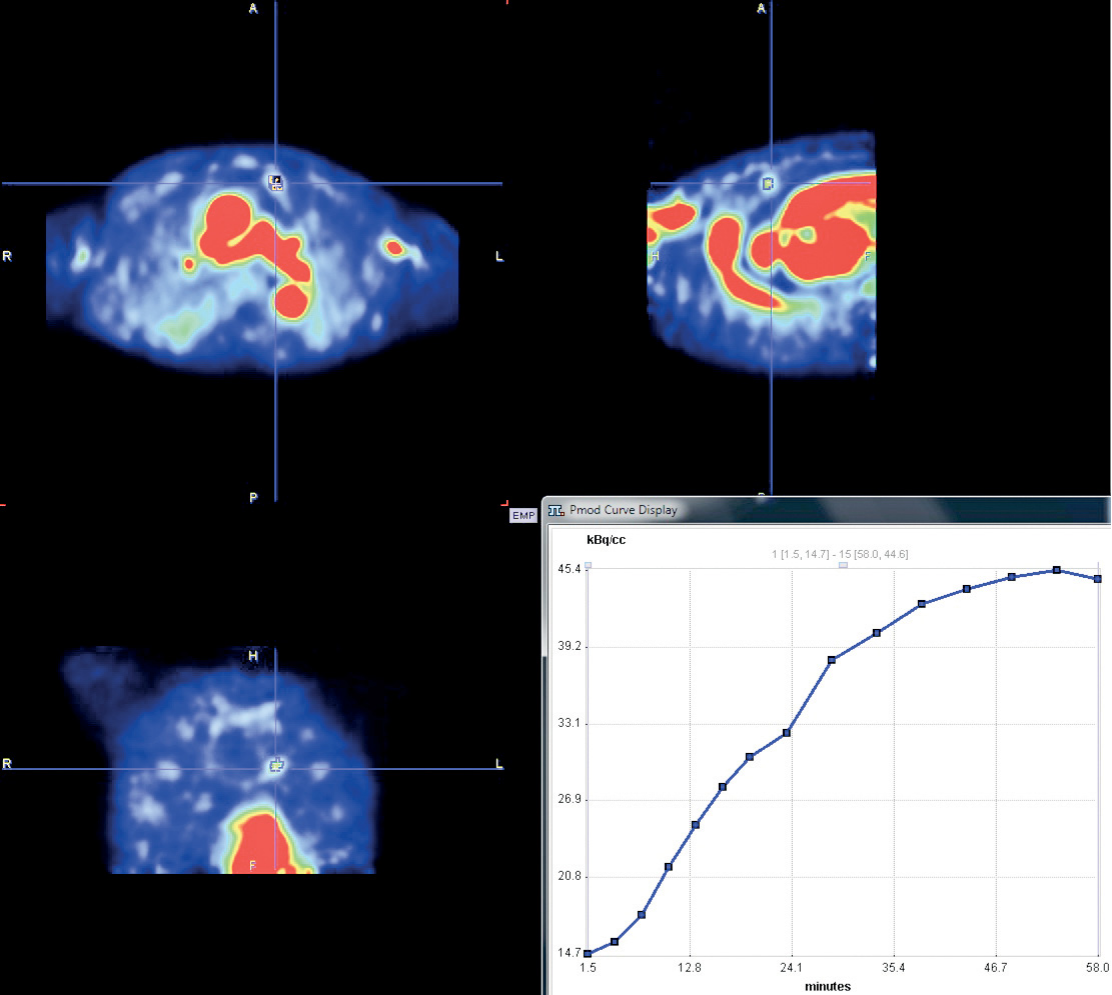
Dynamic PET images of a small pulmonary nodule in the left upper lobe and corresponding time activity curve (TAC) of the nodule displayed by PMOD 3.409 software.

**Figure 2:**
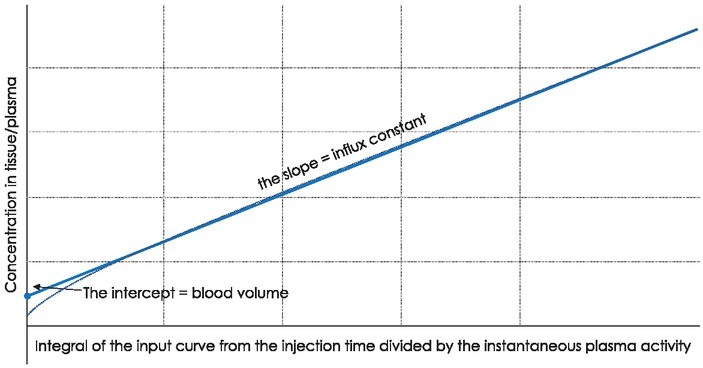
Patlak plot derived from the tissue time activity curve (TAC) and the input function (plasma TAC). The Patlak plot becomes linear after the tracer concentrations in reversible compartments and in plasma are in steady state.

Reconstructed images were imported into PMOD 3.409 software (PMOD Technologies, Zurich, Switzerland) and the input function was determined by placing a spherical volume of interest (VOI) with diameter of 1 cm in the ascending aorta. VOIs were drawn around the pulmonary nodules semi-automatically with a threshold of 50% of the maximum voxel value within the nodule, and then the VOIs were copied to the dynamic imaging sequence to obtain the time activity curves (TACs) (Figure 1). The influx constant *K_i_* (min^-1^ or (ml plasma)*(ml tissue)^-1^*min^-1^)) was determined by Patlak analysis.^35^ The Patlak plot model is a graphical analysis technique based on a 2-tissue compartment model with irreversibly trapped tracer. A mathematical transformation of the tissue compartment and plasma TACs produces a straight line plot which provides information about the blood volume (BV) of the tissue compartment and the exchange rate (*K_i_*) (Figure 2).

#### Conventional PET/CT scan

The maximum standardised uptake value (SUV_max_) was measured for each nodule on conventional FDG PET/CT images. For the semi-quantitative analysis, the mean standardised uptake values (SUV_mean_) were measured of the ascending aorta at the level of the arch, and within the right lobe of the liver. SUV ratios (SUR) were calculated between the nodule SUV_max_, and the SUV_mean_ of the mediastinal blood pool (SUR_BLOOD_) and liver (SUR_LIVER_). Criteria for malignancy were specified as SUV_max_ ≥2.5; SUR_BLOOD_ ≥1.56; SUR_LIVER_ ≥1.12. Qualitative assessment PET features were specified as following: 0 = no visible uptake; 1 = uptake less than mediastinal blood pool; 2 = uptake comparable to mediastinal blood pool; 3 = uptake greater than mediastinal blood pool; 4 = distant metastases. Qualitative specified criteria for malignancy was PET grade ≥ 3.^36^, ^37^ VOIs were placed over the nodules, the ascending aorta at the level of the arch, and within the right lobe of the liver for determination of the SUV_mean_ and SUV_max_ values using OsiriX software (OsiriX, version 8.0.1 64 bit; OsiriX Imaging Software, Geneva, Switzerland).

#### Perfusion CT

Perfusion analysis was performed using Body Perfusion Application on a Vitrea Workstation (Vitrea fX 6.0; Vital Images, Minnetonka, MN, USA). Regions of interest (ROIs) were placed over the pulmonary nodules and contralateral lung parenchyma (diameter range, 7–29 mm) on all perfusion CT images. Arterial input was determined by placing 1 cm ROI over the main pulmonary artery. Time-density graphs were then reviewed and adjustments to start point and end point of the maximum slope were made if needed to define the optimal slope range. Arterial flow perfusion maps overlaying CT images were visually analysed and ROIs were placed over the nodules to obtain the equivalent blood volume parameter calculated by Patlak plot model (BV, expressed in ml per 100 ml) and Arterial Flow (AF, expressed in ml per 100g per minute) using single-input maximum slope model for calculation.

### Statistical analysis

All results were expressed as mean ± standard deviation (SD) unless indicated. *K_i_* and perfusion indices BV and AF of benign and malignant nodules were statistically compared using the nonparametric Mann-Whitney U test. The accuracy of the different techniques and parameters was tested with area under the curve (AUC) in receiver operating characteristic (ROC) analysis with 95% confidence interval (CI). Comparison between the ROCs was performed using DeLongs test. Youdin index analysis was used to derive the optimised cut-point values. Mann-Whitney U test and ROC curve analyses were performed on GraphPad Prism version 8.2.1 for Windows (GraphPad Software, San Diego, CA, USA). Youdin index analysis and nonparametric DeLongs test were performed on MedCalc Statistical Software version 19.8 (MedCalc Software Ltd, Ostend, Belgium; https://www.medcalc.org;2021). A p value < 0.05 was considered statistically significant.

## Results

**Figure 3:**
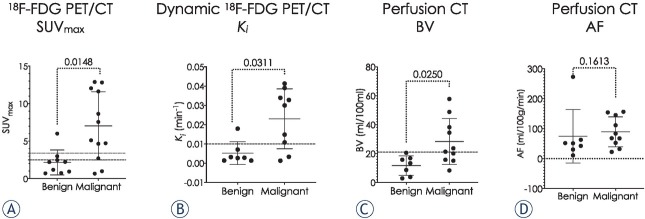
**(A)** standardized uptake value (SUV_max_), **(B)** Dynamic ^18^F-FDG PET/CT influx constant (*K_i_*), **(C)** Perfusion CT parameters blood volume (BV) and **(D)** Average arterial flow (AF) of the benign and malignant nodules.

The demographic data, average nodule size, SUV_max_, metabolic parameter relating to the pulmonary nodules through dynamic ^18^F-FDG PET/ CT, and perfusion parameters through perfusion CT for the benign and malignant nodules are summarised in Table 1 and Figure 3. We analysed 21 soft tissue nodules in 20 patients (male/female = 11/9; mean age ± SD: 65.3 ± 7.4; age range: 50–76 years) with mean nodule diameter ± SD of 20.1 ± 7.5 mm (9–29 mm); mean nodule volume ± SD: 2849 ± 2338.7 mm^3^ (247–9348 mm^3^). 52% of the nodules were located in the upper lung lobes (right upper lobe 7/21, left upper lobe 4/21), 48% were in middle and lower lung lobes (right middle lobe 2/21, right lower lobe 6/21 and left lower lobe 2/21). Final diagnosis was determined after surgical resection in 10 patients, core CT guided biopsy or bronchoscopy in 6 patients, and over 2 years stability on follow up CT imaging in 5 patients.

**Table 1:** The demographic data, average nodule size, standardized uptake value (SUV_max_), metabolic parameter relating to the pulmonary nodules through dynamic ^18^F-FDG PET/CT, and perfusion parameters through perfusion CT for the benign and malignant nodules

	Benign Nodules	Malignant Nodules	p value
Total Number of nodules	9	12	
Number of male patients (%)	5/9 (55 %)	6/12 (50 %)	
Average patient age (years ± SD)	63 ± 7.5	68 ± 6.7	
Average nodule size, range (mm)	18, 9–29	22, 12–30	
Average SUV_max_^18^F-FDG PET/CT ± SD	2.2 ± 1.7	7.0 ± 4.5	0.0148
Number of nodules analysed for dynamic ^18^F-FDG PET/CT	7	9	
Average *K_i_* ± SD (min^-1^)	0.0057 ± 0.0071	0.0230 ± 0.0155	0.0311
Number of nodules analysed for perfusion CT parameters	7	10	
Average BV ± SD (Patlak, ml/100ml)	11.6857 ± 6.7347	28.3400 ± 15.9672	0.0250
Average AF ± SD (ml/100g/min)	74.4571 ± 89.0321	89.2000 ± 49.8883	0.1613

AF = Arterial flow; BV = blood volume; *K_i_* = influx constant; SD = standard deviation; SUV = standardized uptake value

As shown in Table 1 and Figure 3, SUV_max_ derived from the conventional ^18^F-FDG PET/CT and *K_i_* derived from dynamic ^18^F-FDG PET/CT were significantly higher in malignant nodules than in benign nodules. Also, the Patlak model derived BV on perfusion CT was significantly higher in malignant nodules. The difference in AF between the benign nodules and malignant nodules was not statistically significant.

The benign outlier on ^18^F-FDG PET/CT (SUV_max_ = 6.3) and dynamic ^18^F-FDG PET/CT (*K_i_* = 0.0179 min^-1^) was an 18 mm nodule of inflammation and fibrosis (Figure 3A and B). The perfusion CT indices BV and AF in this nodule were relatively low, 3.8 ml/100ml and 51.5 ml/100g/min, respectively (Figure 3C and D). The two malignant outliers on conventional ^18^F-FDG PET/CT and dynamic ^18^F-FDG PET/CT were 12 mm and 16 mm mucinous adenocarcinomas *in situ* (12 mm nodule with SUV_max_ = 0.7 and *K_i_*=0.0015 min^-1^ (BV and AF analysis non-feasible due to respiratory motion artefact); 16 mm nodule with SUV_max_ = 1.0, *K_i_* = 0.0033 min^-1^, BV = 48.8 ml/100ml and AF = 154.1 ml/100g/ min) (Figure 3A and B). The mean CT densities of these two nodules on unenhanced CT images were 16.3HU and 15.9HU, while the mean density ± SD of all benign and malignant nodules analysed was 24.55 ± 12.01 HU. The benign outlier in AF on perfusion CT was a 10 mm perivascular epithelioid cell tumour (PEComa), AF = 272.7 ml/100g/ min (Figure 3D). The BV in this nodule was 20.5 ml/100ml, the ^18^F-FDG PET/CT indices were low, SUV_max_ = 0.7 and the *K_i_* = 0.001 min^-1^.

**Figure 4:**
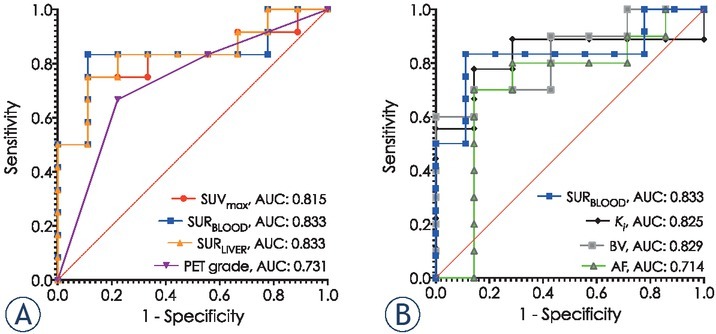
Comparison of AUCs on ROC curves **(A)** SUV_max_, SUR_BLOOD_, SUR_LIVER_, PET grade and **(B)** SUR_LIVER_, K_i_, BV and AF. 95% CI, p values for SUV_max_ / SUR_BLOOD_ / SUR_LIVER_ / PET grade / K_i_ / BV / AF: 0.6264 to 1.000, 0.0157/ 0.6486 to 1.000, 0.0105/ 0.6550 to 1.000, 0.0105/ 0.507 to 0.956, 0.0756/ 0.602 to 1.000, 0.0300/ 0.6322 to 1.000, 0.0248/ 0.4342 to 0.9944, 0.1432.

Table 2 and Figure 4A show diagnostic accuracy of conventional PET/CT derived parameters with pre-specified and derived cut-point values though ROC analysis.^36^, ^37^ SUR_BLOOD_ parameter had overall highest accuracy, however, pairwise comparison of AUCs showed no significant difference (p = 0.5308 *vs*. SUV_max_; p = 1.0000 *vs*. SUR_LIVER_; p = 0.1083 *vs*. PET grade). ROC analysis and diagnostic accuracy for the diagnosis of malignancy by dynamic ^18^F-FDG PET/CT parameter *K_i_*, and perfusion CT indices BV and AF compared to SUR_BLOOD_ are further detailed in Table 2 and Figure 4B. Pairwise comparison of AUCs of SUR_BLOOD_, *K_i_*, BV and AF showed no significant difference in their diagnostic performances (p > 0.1 for all comparisons).

**Table 2:** Comparison of the diagnostic accuracy of different techniques and parameters with pre-specified and derived cut-point values for malignancy

Parameter	Cut-point value/grade		Sensitivity (95% CI)	Specificity (95% CI)	Accuracy
SUV_max_	Pre-specified Derived	≥ 2.5* ≥ 3.4	75.0% (46.8 to 91.1%) 75.0% (46.8 to 91.1%)	66.7% (35.4 to 87.9%) 88.9% (56.5 to 99.43%)	71.4% 81.0%
SUR_BLOOD_	Pre-Derived specified	≥ 1.56	83.3% (55.2 to 97.0%)	88.9% (56.5 to 99.4%)	85.7%
SUR_LIVER_	Pre-specified Derived	≥ 1.12 ≥ 1.65	83.3% (55.2 to 97.0%) 75% (46.8 to 91.1%)	66.7% (35.4 to 87.9%) 88.9% (56.5 to 99.4%)	76.2% 81.0%
SUV grade	Pre-specified & Derived	≥ 3	66.7% (39.0 to 86.2%)	77.8% (45.3 to 96.0%)	71.4%
*K_i_*	Derived	≥ 0.01 min^-1^	77.8% (45.2 to 96.0%)	85.7% (48.7 to 99.3%)	81.3%
BV	Derived	≥ 21 ml/100ml	70% (39.7 to 89.2%)	100% (64.6 to 100%)	82.4%
AF	Derived	≥ 65 ml/100g/min	70% (39.7 to 89.2%)	85.7% (48.7 to 99.3%)	76.5%

* = adding cut-points standardized uptake value (SUV_max_) ≥ 1.75 and ≥ 3.6 for nodules < 12 mm and > 16 mm, respectvely,^37^ resulted in sensitivity, specificity and accuracy of 72.7%, 70.0% and 71.4%, respectively; AF = Arterial flow; BV = blood volume; CI = confidence interval; *Ki* = influx constant; SUR = SUV ratios; SUV = standardized uptake value

## Discussion

Our results demonstrate that the metabolic parameter *K_i_* of dynamic ^18^F-FDG PET/CT and the BV parameter of perfusion CT are significantly lower in benign nodules.

Our study showed that the diagnostic accuracy of the conventional ^18^F-FDG PET/CT was best when semi-quantitative assessment and measuring the uptake ratio of the lung nodule to the mediastinal blood pool with cut-point criteria for malignancy SUR_BLOOD_ ≥1.56 was used. This has been confirmed in a larger multicenter trial by Evangelista *et al*.^36^ Different to the SPUTNIK trial which has shown SUV_max_ to be the most accurate and reproducible technique with a caveat of introducing additional cut-point values altered according to the nodule size, we did not see significant improvement in diagnostic accuracy when replicating the multiple cut-points in our group of nodules (see * in Table 2).^37^

The accuracies of the new metabolic parameter *K_i_* and perfusion parameter BV were not significantly different to the conventional ^18^F-FDG PET/ CT. The derived *K_i_* cut-point for malignancy was ≥0.01 min^-1^ resulting in sensitivity/specificity/accuracy of 77.8%/85.7%/81.3%, respectively. This is in good agreement with *K_i_* cut-point ≥0.014 min^- 1^ reported in the study by Huang *et al*. (n = 35).^26^ The derived BV cut-point value of ≥21 ml/100ml for malignancy showed comparable diagnostic accuracy to conventional and dynamic ^18^F-FDG PET/ CT parameters. The high specificity of BV demonstrated in our nodules would need to be confirmed in larger studies.

The benign outlier on dynamic ^18^F-FDG PET/CT with a high *K_i_* parameter histopathologically represented inflammation (Figure 3B). Higher metabolic activity is not only a feature of malignant cells, it can be observed in inflammatory nodules due to increased glucose metabolism in granulocytes and macrophages in a range of diseases, including fungal and necrobiotic rheumatoid nodules, sarcoidosis, tuberculosis, and other granulomas.^38^, ^39^ Dual time PET/CT did not prove to be useful for differentiating benign and malignant pulmonary nodules with an SUV_max_ less than 2.5 in regions with high prevalence of granulomatous disease.^40^, ^41^ Huang *et al*. showed that dynamic ^18^F-FDG PET/ CT is valuable in differentiating benign from malignant pulmonary nodules with the potential to differentiate malignant from granulomatous disease.^26^ Our study showed limited diagnostic accuracy of the dynamic ^18^F-FDG PET/CT in assessing inflammatory nodules.

The malignant outliers on dynamic ^18^F-FDG PET/CT with low *K_i_* parameters were histopathologically mucinous adenocarcinoma *in situ*. Other malignant nodules in which low metabolic activity can be measured on ^18^F-FDG PET/CT are minimally invasive adenocarcinoma, carcinoid, and lung lymphoma.^38^,^42^ Another important finding was that both malignant nodules with low metabolic activity were of lower CT density analysed on the initial perfusion CT images but also appreciable on low-dose CT scan of PET/CT examination. Further studies on low density lung nodules are needed for evaluation of using lower cut-point values for malignancy in conventional and dynamic PET/ CT. Malignant lung nodules with low CT density and measuring less than 1 cm are known to have low metabolic activity on conventional ^18^F-FDG PET/CT.^43^, ^44^ Berger *et al*. have reported up to 41% of lung lesions to be false-negative on conventional ^18^F-FDG PET/CT in analysis of 25 mucinous, hypocellular lung lesions (3/9 false negative lesions were ≤ 2 cm, range, 1–5 cm).^45^ Our study showed a limited diagnostic accuracy of the dynamic ^18^F-FDG PET/CT in assessing low density malignant pulmonary nodules with *K_i_* cut-point set at 0.01 min^-1^.

Dynamic enhancement CT studies help identify false positive results in both inflammatory and infective conditions, and sometimes in benign vascular tumours.^46^, ^47^ The perfusion CT parameters for the inflammatory nodule in our study were low and indicative of a benign lesion despite high metabolic activity on ^18^F-FDG PET/CT. We have shown that the parameters of perfusion CT of both malignant nodules with low metabolic activity were higher than the BV and AF in benign nodules. Therefore, our findings indicate parameters of perfusion CT may aid in the identification of benign nodules with high glucose metabolic activity and in the identification of malignant nodules with low glucose metabolic activity. Ohno *et al*. have shown that perfusion CT is more specific and accurate than conventional ^18^F-FDG PET/CT.^24^,^29^ Our study on a small sample of cases suggests that perfusion CT also performs better than dynamic ^18^F-FDG PET/CT.

The AF parameter of the perfusion CT obtained by the maximum slope method was not significantly different between benign and malignant nodules. Benign nodules had a lower AF parameter value than malignant nodules overall with one significant benign outlier with markedly high AF. Histopathologically, this represented an extremely rare ‘light cell’ or ‘sugar type’ PEComa. There are only about 50 cases of this neoplasm described in the literature.^48^, ^49^ PEComas are more commonly found as angiomyolipomas in the kidneys, or as lesions in the retroperitoneal space, gastrointestinal tract, or uterus. Only 7 cases of malignant pulmonary PEComa have been reported.^50^ A case report of a benign pulmonary PEComa showing early wash-in enhancement with an early washout pattern of a malignant lesion on perfusion CT has been reported by Kim *et al*.^51^ Despite a markedly high AF, the PEComa had a BV just under the cut-point value for malignancy and a low metabolic parameter *K_i_* of dynamic ^18^F-FDG PET/CT. The BV parameter in combination with low *K_i_* parameter proved to be more reliable for defining this extremely rare histological type of a pulmonary nodule.

Our study has limitations. This pilot study was performed in a small sample of patients and appropriately powered studies will be required for further validation. The mean nodule size was 18 mm for benign and 22 mm for malignant nodules, which would not normally be referred for imaging follow-up. The BTS and Fleischner Society recommended lower thresholds for nodule follow up (5 mm and 6 mm, respectively). More novel reconstruction methods in PET/CT such as specific point spread function (PSF) are enabling better spatial resolution and enable its use in 6 mm pulmonary nodules.^52^

Perfusion CT is quite demanding on patients with a prolonged breath-hold, which limits the availability of reliable data in some patients. All 3 nodules in which analysis was non-feasible due to the significant breathing artefact were near the diaphragm (2 in the right lower lobe and 1 in the right middle lobe). Segmentation of the pulmonary nodules on image analysis is restricted when the images were affected by respiratory motion artefact, especially in small nodules which were also abutting the chest wall or mediastinal structures. Some authors recommend quiet breathing during the perfusion CT scans but this is only acceptable in larger lung masses.^53^ There is a need for further optimisation of nodule segmentation and advanced image registration techniques that allow accurate assessment of pulmonary nodules without need for long breath-hold.^23^,^54^ The effective radiation dose for dynamic ^18^F-FDG PET/CT was around 8 mSv and for perfusion CT around 20 mSv. The radiation dose for perfusion CT can be improved by reducing the field of view from 16 cm to the nodule only and reducing tube voltage in smaller size patients.^55^

Potential increase in the demand for these not widely available novel dynamic imaging studies would consequently put additional strain on the imaging departments with increased demand for scanner time, funding and training of the staff. Limited capacity for a wider use of the dynamic imaging in lung nodules could be overcome by developing systems of identification of nodules with highest diagnostic benefit from dynamic imaging. A multicentre prospective cohort observational study initiated in 2016 is set to assess the performance and the cost-effectiveness of the dynamic CT and PET/CT in the characterisation of solitary pulmonary nodules.^56^

The small sample size limits the assessments of accuracy in our study. However, on this small sample we showed increase diagnostic improvement in the accuracy of diagnosis in both dynamic studies when compared to the conventional ^18^F-FDG PET/ CT. Specificity in *K_i_* and BV on our small sample size were higher at the estimated threshold values of 0.01 min^-1^ and 21 ml/100ml, respectively. This would need to be confirmed in larger studies.

Early identification of a lung nodule as benign or malignant by analysing its metabolic and perfusion parameters could reduce the need for CT to monitor lung nodule size, thereby reducing the number of CT scans required. It could also reduce the need for CT guided biopsy or other invasive procedures. Patients with malignant lung nodules could thus be identified more quickly and referred for radical treatment. With our study, we have demonstrated the potential of perfusion CT. The BV parameter assessed by perfusion CT was not only significantly lower in benign nodules, it also aided in correctly characterising the metabolically active inflammation, hypervascular benign PEComa and low density malignancy.

In conclusion, this study demonstrated the feasibility of dynamic ^18^F-FDG PET/CT and CT perfusion studies in differentiating benign and malignant pulmonary nodules. The dynamic ^18^F-FDG PET/CT and perfusion CT derived blood volume can assist to differentiate benign and malignant lung nodules and in indeterminate cases, a combined approach can be helpful.
